# A Cross-Country Examination on the Fear of COVID-19 and the Sense of Loneliness during the First Wave of COVID-19 Outbreak

**DOI:** 10.3390/ijerph18052586

**Published:** 2021-03-05

**Authors:** Gianluca Lo Coco, Ambra Gentile, Ksenija Bosnar, Ivana Milovanović, Antonino Bianco, Patrik Drid, Saša Pišot

**Affiliations:** 1 Department of Psychology, Educational Science and Human Movement, University of Palermo, Viale delle Scienze, Edificio 15, 90128 Palermo, Italy; ambra.gentile91@gmail.com (A.G.); antonino.bianco@unipa.it (A.B.); 2 Faculty of Kinesiology, University of Zagreb, 10000 Zagreb, Croatia; ksenija.bosnar@kif.unizg.hr; 3 Faculty of Sport and Physical Education, University of Novi Sad, 21000 Novi Sad, Serbia; i.a.milovanovic@gmail.com (I.M.); patrikdrid@gmail.com (P.D.); 4 Institute for Kinesiology Research, Science and Research Center Koper, 6000 Koper, Slovenia; sasa.pisot@zrs-kp.si

**Keywords:** COVID-19, fear of COVID-19, loneliness, cross-country, psychosocial distress

## Abstract

The aim of the current study is to examine gender, age. and cross-country differences in fear of COVID-19 and sense of loneliness during the lockdown, by comparing people from those countries with a high rate of infections and deaths (e.g., Spain and Italy) and from countries with a mild spread of infection (e.g., Croatia, Serbia, Slovakia, Slovenia, and Bosnia and Herzegovina). A total of 3876 participants (63% female) completed an online survey on “Everyday life practices in COVID-19 time” in April 2020, including measures of fear of COVID-19 and loneliness. Males and females of all age groups in countries suffering from the powerful impact of the COVID-19 pandemic reported greater fear of COVID-19 and sense of loneliness. In less endangered countries, females and the elderly reported more symptoms than males and the young; in Spanish and Italian samples, the pattern of differences is considerably more complex. Future research should thoroughly examine different age and gender groups. The analysis of emotional well-being in groups at risk of mental health issues may help to lessen the long term social and economic costs due to the COVID-19 outbreak.

## 1. Introduction

By the mid of January 2020, the Chinese government had quarantined the city of Wuhan (11 million inhabitants) and subsequently extended the measure to Hubei province (60 million inhabitants) to contain the Coronavirus Infectious Disease 2019 (COVID-19) epidemic. Since that time, there has been a progressive spread of the virus throughout the world, with 24,854,140 reported infections and 838,924 deaths attributed to COVID-19 by 30 August 2020 [1]. On 11 March 2020, the World Health Organization (WHO) declared a state of pandemic. Quarantine (i.e., the segregation of one or more healthy people inside their own homes, to prevent infection and the virus spreading) was considered one of the most helpful measures in containing the infection. Most countries issued varying degrees of “shelter-in-place” orders [2] and almost one-third of the global population has faced some form of quarantine [1] due to the COVID-19 outbreak in the last few months. However, there is evidence that undergoing quarantine can have detrimental effects on people’s psychological health [3], with anxiety, anger, insomnia, and somatic symptoms, mainly due to the loss of freedom, the separation from loved ones, uncertainty over the disease, and shortage of everyday supplies.

To date, there is some evidence showing the negative impact of the COVID-19 pandemic on psychological well-being [4,5,6]. One of the first surveys, which was conducted in China during the lockdown, showed that more than 50% of participants rated the psychological impact of COVID-19-related restrictions as moderate or severe [7], with greater difficulties associated with the effects of COVID-19 pandemic on daily life and social and work activities [8,9,10]. Of course, this negative impact is even greater for healthcare professionals tackling this global crisis [11], with a considerable proportion of workers reporting symptoms of depression, anxiety, and stress [12]. The negative psychological effects of the COVID-19 pandemic on the individual’s mental health states were further confirmed in studies from several Western countries [13,14,15].

As COVID-19 continues to spread, so does the research on people’s experience of fear during the pandemic. Fear of personal infection or infecting loved ones is common among people exposed to any infectious disease outbreak [3,10], and it is worth carrying out a specific examination of the characteristics of the fear of COVID-19. Globally, more than 72 million people have contracted the virus infection, and 1.6 million have died (by first week of December 2020). Thus, it is likely that the high mortality rates due to COVID-19 have negatively impacted on the individual’s feelings of fear of contagion and anxiety throughout all countries of the world. In the current study, we will focus on a cross-country examination of the COVID-19 outbreak and on the fear of COVID-19, by differentiating between European countries that reported a powerful impact of the infection (e.g., Italy and Spain) and those that reported a mild impact (e.g., Croatia, Serbia, Slovakia, Slovenia, and Bosnia and Herzegovina) during the first wave of the pandemic (see Supplemental Figure S1). More specifically, during the time lag of the current study (15–28 April 2020), there has been a reported cumulative 199,414 infected and 26.977 deaths in Italy (329 infected rate /9.8 death rate), and 213,095 infected and 23.822 deaths (455/11.9) (Infected rate (infected/100.000 inhabitants, death rate (deaths/100.000)) in Spain. In only the time of the online survey, they both witnessed over 10.000 deaths. These numbers are higher than those that were officially reported in Croatia (49.7/0.7) Slovenia (67.7/1.3), Serbia (94.7/0.8), Slovakia (15.8/0.2), and Bosnia and Herzegovina (47.7/0.6). Both Italy and Spain have applied emergency epidemiological measures: first quarantine, and then total lockdown. The other countries covered by this research adopted mild restrictions, i.e., the introduction of a state of emergency, with curfew (Serbia, Bosnia and Herzegovina), the introduction of a state of emergency without curfew (Slovenia, Slovakia), and the “closure of public life” (Croatia). Given the aforementioned differences, not only in numbers of citizens infected/deceased, but also in the nature/type of epidemiological emergency measures, the investigation of differences regarding the negative consequences of the COVID-19 pandemic seems worthwhile.

Although fear is an adaptive response in the presence of danger, it has been suggested that the construct of fear of COVID-19 should be examined within an integrated complex model [16]. For example, fear of infection can trigger healthy behaviors or, on the contrary, prompt anxiety about health. Concerns and fears about one’s own health and the well-being of one’s own beloved ones (particularly the elderly or people suffering from any physical illness) can exacerbate feelings of anxiety. If these concerns are prolonged over time, they may increase the risk of serious mental health conditions, including anxiety disorders, stress, and trauma-related disorders [17]. Moreover, feelings of uncertainty about the future and the lack of an effective vaccine may have led people to heighten their fear of COVID-19 during quarantine. To date, some new tools for the assessment of the Fear of COVID-19 have been developed [18,19,20] to provide healthcare professionals with a valid measure for monitoring fear and anxiety of individuals during the COVID-19 crisis [18,21]. Previous research showed a significant association between the fear of COVID-19 and the most widely-recommended strategies to control the spread of COVID-19, such as spatial distancing and handwashing [22,23]. People with an excessive fear of the infectious outbreak are more likely to report greater psychosocial distress, whereas people showing little anxiety are more likely to disregard the physical distancing [20,24].

An important step towards understanding the critical characteristics of this construct is to examine the cross-country similarities and differences in fear of COVID-19. Although there is some evidence to suggest that fear of COVID-19 may be concentrated in those regions with the highest reported COVID-19 cases [25], there has been limited research as to whether fear may differ in those European populations subjected to a high or limited impact of the infection and to policies of strict restriction. Moreover, the association between fear of COVID-19 and social isolation during the lockdown needs to be further investigated in cross-cultural research. To date, the link between people’s experience of fear of COVID-19 and feelings of loneliness has received little research attention. Although physical distancing measures have been critical in containing the rate of infection, there is concern that limits on social activities and restrictions on in-person social contacts may increase feelings of loneliness [26,27]. Prior research on the experience of loneliness in response to the social restrictions due to the COVID-19-related quarantine reported mixed findings. For example, it was shown that being under a stay-at-home order was associated with greater loneliness and health anxiety. However, the higher perceived impact of COVID-19 on participants’ daily life was significantly associated with higher perceived social support and lower loneliness [14]. Moreover, a recent longitudinal study [27] showed that although people perceived an increased absence of social connections during the initial stages of the COVID-19 outbreak, they did not feel more isolated in response to the implementation of social distancing measures.

To the best of our knowledge, no previous research has examined the link between fear of COVID-19 and feelings of loneliness during the lockdown transversely across countries. It is likely that lockdown measures have resulted in worsening individual’s sense of loneliness and fear of COVID-19. Although some studies showed that individuals who felt lonely in the pandemic reported symptoms of anxiety and depression [13,28], and that greater emotion regulation difficulties and depression may be risk factors for loneliness [29], interplay remains unknown between feelings of loneliness and fear of COVID-19 in countries facing varying levels of the spread of infection as well as different home-confinement policies.

The present study examines individuals’ experience of fear of COVID-19 and loneliness in response to physical distancing and restriction measures undertaken to contain the outbreak of COVID-19 in different countries. More specifically, this study aims to examine potential cross-country differences in the measures of fear of COVID-19 and loneliness across two groups of European countries subject to varying impact of the COVID-19 pandemic (e.g., with regard to the number of deaths and measures of total lockdown). We hypothesize that fear of COVID would be associated with loneliness during the pandemic and can represent top stressors. Moreover, in line with prior studies [13,28], we expected that countries reporting a high death and infection rate would display a higher fear of COVID-19, associated with feelings of loneliness, compared to countries reporting a low infection and death rate in the midst of the pandemic. We also aim to examine gender and age group differences across countries. We do expect gender differences in fear of COVID-19 and loneliness, and it was hypothesized that females would report more fear of COVID-19 and would feel lonelier than males, in accordance with previous research [27,29]. Finally, we expected that the elderly would feel lonelier and would also display greater fear of COVID-19 than the young in all countries [6,13].

## 2. Materials and Methods

### 2.1. Participants and Data Collection

The sample consisted of 3876 participants (1422 males, 2442 females) from 7 European countries (Italy, Spain, Bosnia and Herzegovina, Croatia, Serbia, Slovakia, Slovenia), whose ages ranged between 18 and 82 years (*M* = 31.94; *SD* = 12.02). The majority of participants described themselves as female (*N* = 2442, *M*_age_ = 31.88 years; *SD* = 12.96), 1422 described themselves as male (*M*_age_ = 32.05 years; *SD* = 13.14), and 12 described themselves as other gender (e.g., transgender, bigender, non-binary). However, given the very low number in this grouping (0.3%), in the current study, we limited data analyses to men and women.

Recruitment of participants was designed as an online survey with a general invitation to participate. Participants could “respond,” i.e., choose to participate, without receiving incentives. From the point of view of sampling within the consortium of the 7 countries that conducted the study, the first target group was students of faculties of the consortium and then their wider social networks. Participants were invited to participate in the survey with personal transmission of the questionnaire via individual e-addresses databases and posting the link to the questionnaire on social networks, official webpages of partners’ organizations, and local on-line newspapers.

Participants completed a 22-item online survey “Everyday life practice in COVID-19 time” during the restriction time for COVID-19 pandemic (see Supplemental information), from 15 April 2020 to 28 April 2020 [30]. Participants had to be 18 years or older and living in the European countries indicated. They were categorized into four age groups: emerging adults (between 18 and 25 years old), young adults (between 26 and 39 years old), middle-aged adults (between 40 and 60 years old), and older adults (60 years or older) (see Table 1). However, due to the COVID-19-related restrictions and the limited recruitment window (14 days) we were able to conduct a non-probability sample. All materials and procedures were reviewed and approved by the consortium of six partners from Science and Research Centre Koper, Slovenia; Faculties of Sport at University of Novi Sad, Serbia; University of Palermo, Italy; University of Zagreb, Croatia; University of Presov, Slovakia; and University of Cadiz, Spain. The study was conducted in accordance with the ethical standards of the Declaration of Helsinki, and all participants signed statements of informed consent to participate in this study. The Ethics Committee of the University of Novi Sad (Nr. 46-06-02/2020-1) approved this study prior to data collection. Each institution of the participating countries agreed to move forward with the study under the Institutional Review Board approval of the University of Novi Sad. Participants were informed that all data would have been processed and managed by the legislation for the protection of personal data and the General Data Protection Regulation (GDPR). They were able to leave the questionnaire at any stage before the submission process. Only surveys with completed mandatory questions were taken for further analysis.

### 2.2. Measures

The survey was made up of socio-demographic questions (revealing age, gender, education, and nationality), the Fear of COVID-19 Scale [18], and the Three-Item Loneliness Scale [31]. The questionnaires were translated and back-translated to ensure that the wording was appropriate for Spain, Bosnia and Herzegovina, Croatia, Serbia, Slovakia, and Slovenia. The study was conducted in line with some recommendations by Swami and Barron [32] to ensure semantic equivalence. In the first step (forward translation) the original questionnaire was translated into the target languages by two mother tongue translators. Each translator produced an independent translation, and all participated to a synthesis meeting. For back translation, two separate native English-speaking translators independently translated the synthesized version of the target questionnaire into English. The forward and back translations were reviewed by a research committee with language professionals and methodologists, in order to make final semantic adjustments and produce the final version of the measure.

### 2.3. Fear of COVID-19 Scale

Participants completed the Fear of COVID-19 Scale by Ahorsu et al. [18], which consists of 7 items with answers on a 5-point scale, from completely disagree to agree. It was constructed considering existing scales on fears, expert evaluations, and interviews, and it shows very good psychometric properties. Specifically, it shows stable psychometric properties across countries, with a good reliability (Cronbach alphas: Italy, 0.86; Spain, 0.87; Bosnia and Herzegovina, 0.89; Croatia, 0.85; Serbia, 0.85; Slovakia, 0.83; Slovenia, 0.85).

### 2.4. Three-Item Loneliness Scale

Loneliness was measured by the 3-item Loneliness Scale by Hughes et al. [31]. It consists of three items determining lack of companionship, the feeling of being left out, and the feeling of being isolated from others, measured on the frequency Hardly Ever, Some of the Time, and Often. For the purposes of the present study, the items were treated as three different indicators of feelings of loneliness.

### 2.5. Data Analysis

Descriptive statistics of the total result for the Fear of Covid-19 Scale and items from the Three-Item Loneliness Scale were calculated on the total sample and subgroups regarding gender, age, and country. Correlational analysis through Pearson’s *r* was performed to see whether loneliness items and fear of COVID-19 were interrelated. Countries were divided into two groups, with, specifically, the most endangered, Italy and Spain (C2), in one group and Slovenia, Croatia, Serbia, Slovakia, and Bosnia and Herzegovina in the other group (C1).

Establishing significant multivariate differences of two or more groups was tackled by discriminant analysis [33,34]. Subsequently, the canonical multi-group discriminant analysis of groups defined by age, gender, and country was carried out on the total result of the Fear of COVID-19 Scale and items from the Three-Item Loneliness Scale, by using the Discriminant Function Analysis procedure described by Jennrich [35] in STATISTICA (version 13.0, TIBCO, Palo Alto, CA, USA). The results of multivariate and multiple-group discriminant analysis are (1) the number of significant discriminant functions, (2) the identification of variables defining each discriminant function, and (3) the mapping of the groups in the space defined by discriminant functions [36]. The significance of the first and subsequent discriminant functions was tested by Wilks’ lambda values at the level of statistical significance *p* < 0.01. Standardized discriminant coefficients and correlations of independent and discriminant variables were determined. The means for the discriminant functions by group (namely, group centroids) were computed; centroids were represented in three-dimensional Cartesian space.

## 3. Results

### 3.1. Preliminary Results

Descriptive statistics are summarized in Supplement Tables S1 and S2. Twelve participants who identified as being in the “other” gender category were excluded. Therefore, the total sample size was *N* = 3864 for further testing on the Fear of COVID-19 and the Loneliness scales. Fear of COVID-19 and loneliness items (lack of companionship, feeling left out, and feeling isolated) were significantly correlated at *p* < 0.01. Independent variables distributions are significantly different from the norm because of skewness, but this should not invalidate the discriminant analysis [37,38].

### 3.2. Discriminant Analysis

Canonical discriminant analysis of groups defined by age, gender, and country for the Fear of COVID-19 and the Three-Item Loneliness Scale resulted in three significant discriminant functions (see Table 1), whose discriminant coefficients are represented in Table 2. The first discriminant function is predominantly defined by the result of Fear of COVID-19 (standardized discriminant coefficient = 0.963; correlation with discriminant function = −0.896); feeling that the lack of companionship contributes to a lesser extent (standardized discriminant coefficient = −0.351; correlation with discriminant function = −0.325). The second discriminant function is mainly defined by feeling isolated from others (standardized discriminant coefficient = −0.709; correlation with discriminant function = −0.926) and the tendency to feel the lack of companionship more (standardized discriminant coefficient = −0.313; correlation with discriminant function = −0.689). The third discriminant function is determined by feeling left out (standardized discriminant coefficient = 1.233; correlation with discriminant function = 0.74); partial contributions of two further measures of loneliness are also detected, but to a much lesser extent (standardized discriminant coefficients of feeling the lack of companionship and feeling isolated are −0.489 and −0.430, respectively).

**Figure 1:**
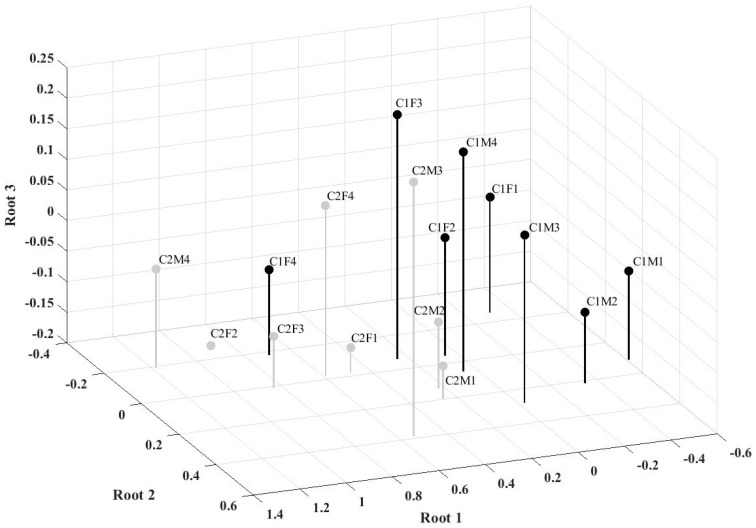
Centroids representation on first, second, and third discriminant functions. Note: Country: C1 and C2. Gender: M, F. Age groups: 1—emerging adults 18–25 years, 2—young adults 26–39 years, 3—middle-aged adults 40–59 years, and 4—elderly of 60 years and more.

Regarding the negative side of the first discriminant function (See Figure 1), described mainly by the lower level of Fear of COVID-19, three centroids of groups lie in less endangered countries (C1); the results of males from C1 countries are either negative or near-zero, values rising from the youngest group upwards (Table 3, Figure 1).

The centroids of female groups from C1 countries also show that fear increases with age; compared with males, female centroids are shifted to higher values and only the youngest group centroid is positioned on the negative side of the function. All the centroids of groups from Italy and Spain (C2), on the first discriminant function, are on the positive side of the function; they do not show the same regularity as groups from less endangered countries. The first three age groups of males have lower values then corresponding female groups, but the eldest males have the highest centroid value on the first function. The emerging-adult female group has the lowest value, whereas the highest value is in the young adult female group.

The centroids of groups from less endangered countries (C1), on the second discriminant function, show that females feel isolated from others more often and feel the lack of companionship more than males. Three female groups from Italy and Spain (C2) have near-zero values and only the young adult female group has centroid on the negative side of function; males from the first three age groups have centroids on the positive side of the function, and the eldest male group has the centroid on the negative side of the function.

The third discriminant function is defined by the feeling of being left out. In the groups from less endangered countries (C1) only elderly males and middle-aged females have centroids on the positive side of the function, the others have near-zero values. Seven groups from Italy and Spain (C2) have near-zero or centroids on the negative side of the function; only the group of middle-aged males felt they were being left out more often.

## 4. Discussion

The present study examined cross-country differences concerning the fear of COVID-19 and loneliness due to the varying degrees of outbreak severity. Our results suggest that both males and females in European countries, in April 2020, suffering from a powerful impact of the COVID-19 pandemic (i.e., Italy and Spain) reported higher fear of COVID-19 and sense of loneliness than those in countries with a lesser spread of the virus. Consistent to our hypotheses, it is likely that the higher number of infections and deaths in the first months of the first wave of the pandemic and the strict shelter-in-place orders in Italy and Spain could have fostered heightened levels of fear of COVID-19 and feelings of loneliness. Moreover, from the analyses, it resulted that people who had a high level of fear of COVID-19 tended to suffer loneliness to a lesser extent and those feeling more isolated also tended to feel the lack of companionship. However, discriminant analysis showed that this pattern of results should be examined in more detail by considering the different age and gender groups.

As expected, from the analysis of the centroids, in countries with low death rates and mild social restrictions (C1), both the emerging adults and young adults did not show a high level of fear, but felt the lack of companionship, especially in the case of men; at the same time, middle-aged and older women showed a higher level of fear and lower lack of companionship. Conversely, people from high death rate countries and harsh restrictions (C2) experienced a higher fear of COVID-19, without feeling the lack of companionship. Furthermore, middle-aged men and young adult women felt both the lack of companionship and being left out. These results are not surprising if we consider that during the time-lag of the survey, Italy and Spain underwent strict restrictions and lockdown, which could have impacted on the people’s sense of loneliness. Moreover, Italy and Spain registered over 10,000 deaths in the two weeks of the survey alone, while Bosnia and Herzegovina, Slovenia, Slovakia, and Serbia had 160 deaths in total. Furthermore, previous data from the USA also showed that fear appeared to be concentrated in regions with the highest reported COVID-19 cases [25].

Regarding the influence of gender, our results confirmed that women reported greater fear than men both in C1 and C2 countries. This finding is consistent with literature showing that females may be more vulnerable to developing psychosocial distress during the pandemic [6,8]. Research on the impact of COVID-19 pandemic on men’s and women’s well-being treated separately is still scarce and there is a need to tackle gender equality in any decision making for the COVID-19 [39]. The findings of the current study suggest that the discriminant functions can be used to identify sub-groups at high risk of distress during the COVID-19 pandemic. The elderly females from countries with low death rate could be considered a group at moderate risk of excessive fear of COVID-19 and lack of companionship. Given the mild restrictions imposed in these countries, this vulnerable group could be supported by regular exercising and maintaining a healthy diet pattern to help prevent symptoms of stress during the pandemic.

Of note, in Italy and Spain, older men (who are at a higher risk of COVID-19 complications) represent a class of individuals at risk of a high fear of COVID-19 and feelings of social isolation. From a policymaking perspective, more attention should be paid to these vulnerable groups by enhancing on-line health services and support. Moreover, these vulnerable groups should be helped in avoiding potentially false reports and continually checking COVID-19 related news, in order to alleviate their feelings of fear and anxiety.

The COVID-19 outbreak is likely to worsen the individual’s perception of loneliness by reducing social interactions and contacts [29]. Given that loneliness is a risk to physical and mental health, there has been a call for a public health framework to tackle loneliness during COVID-19, especially in older adults [40,41]. Our results showed that the older male group in Italy and Spain felt isolated from others and felt the lack of companionship more often. They also reported a higher fear of COVID-19 than other age groups. Thus, they may be identified as a sub-group at high risk of social distress during the COVID-19 pandemic [40]. Overall, the results of this study, which was conducted during the first wave of the COVID-19 pandemic, indicate multiple correlations between fear of COVID-19 and feelings of loneliness, with socio-demographic characteristics of individuals on the one hand and epidemiological emergency measures at the state level on the other. Therefore, they point to the need for more detailed research, with a focus on gender, generation, or socio-economic groups. For example, a cross-cohort study from the UK reported that young adults, people with a lower education and income, and people living alone had a higher risk of being lonely [42]. Further research is necessary to examine whether the accumulation of multiple risk factors can impact on loneliness levels across different European countries. Physicians could help lonely adults to use social services and community-based organizations, and support them in alleviating loneliness and addressing essential needs [43]. Our results showed that females in C1 countries felt isolated from others and with a lack of companionship more often than males. It could be speculated that in countries with mild social restrictions following the COVID-19 outbreak, women were more fully engaged with demanding family activities than men and had less opportunities for social interactions, thus feeling lonelier at this difficult time.

### Strengths and Limitations of the Study

The main strength of the current study is that we examined the impact of the COVID-19 outbreak on fear and the sense of loneliness in a large sample of populations from different European countries. This study also has several limitations. First, the study relies on cross-sectional data, which were collected during the first wave of the pandemic. Further research is needed to examine how the levels of fear of COVID-19 and loneliness changed over time. Second, limitations of the study exist in terms of the self-selective nature of participation in this online study. Furthermore, the recruiting per country resulted as unbalanced. There is evidence that disadvantages of online and single wave approach are a low control over the sample and response rates from low to modest, which can result in an unbalanced structure of the sample [44,45,46]. Further research is necessary to replicate these findings with balanced and representative samples of the general population. Moreover, well-educated people are more likely to participate in an on-line survey than the less-educated, as confirmed by Smith [47], and people of a low socioeconomic status might not be provided with the internet and IT technology. Third, the fear of COVID-19 represents a novel construct and the importance and validity of this variable remains unknown [16]. Moreover, the fear of COVID-19 scale was not fully validated for some of the languages used in the current study and further research is warranted to test its cross-country measurement invariance. Finally, due to the demands of social desirability, using self-report measures may not reflect people’s real opinions and feelings [47].

## 5. Conclusions

Overall, our results show that people from European countries with a high number of infections and deaths during the COVID-19 pandemic reported different levels of fear and feelings of loneliness than people from countries with very low death and infection rates. Our findings support calls for the countries involved to monitor over time the long-term effects of the COVID-19 pandemic on the individual’s levels of fear and loneliness, given the rise of infections and deaths in the second half of 2020. Moreover, our results highlight the fact that future research on the negative health consequences of the COVID-19 pandemic should examine different age and gender groups separately in order to assess which groups might be more vulnerable and, consequently, to take actions to help those at most risk. Analysis of emotional well-being in groups at risk (with mental health issues), may help to lessen the long term social and economic costs due to the COVID-19 outbreak, and integrate behavioral health expertise into public health responses to the pandemic [48,49,50].

Future research on the negative health consequences of the pandemic can build on the cross-country studies that adopted the fear of COVID scale in several European countries, such as Italy, Spain, Israel, Norway, and Russia [51,52,53,54,55], which have consistently supported the importance of assessing the fear of COVID as a relevant clinical outcome among the general population, in order to assist decision-makers and health practitioners to screen the most vulnerable groups.

## Figures and Tables

**Table 1:** Discriminant Analysis results.

Roots Removed	Eigenvalue	Canonical Correlation	Wilks’ Lambda	Chi-Squared	df	*p*
0	0.16	0.37	0.82	776.60	60	<0.001 ***
1	0.04	0.18	0.95	203.01	42	<0.001 ***
2	0.01	0.11	0.98	69.44	26	<0.001 ***
3	0.01	0.08	0.99	24.94	12	0.02 *

(*** *p* < 0.001; ** *p* < 0.01; * *p* < 0.05).

**Table 2:** Standardized discriminant coefficients and correlations with discriminant functions.

Items	Root 1		Root 2		Root 3	
	S	F	S	F	S	F
The Fear of COVID-19 Scale	0.963	0.896	−0.239	−0.405	−0.174	−0.048
How often do you feel that you lack companionship?	−0.351	−0.325	−0.313	−0.689	−0.489	−0.214
How often do you feel left out?	−0.028	−0.056	−0.049	−0.653	1.233	0.740
How often do you feel isolated from others?	−0.141	−0.155	−0.709	−0.926	−0.430	0.060

Note: S—Standardized discriminant coefficient; F—correlation with discriminant function.

**Table 3:** Centroids distinguished according to country (C1 and C2), gender (M, F), and age (1—emerging adults 18–25 years, 2—young adults 26–39 years, 3—middle-aged adults 40–59 years and 4—elderly of 60 years and more).

	Countries 1 (C1)			Countries 2 (C2)		
	Root 1	Root 2	Root 3	Root 1	Root 2	Root 3
M1	−0.587	0.141	−0.047	0.281	0.226	−0.138
M2	−0.316	0.244	−0.075	0.252	0.164	−0.083
M3	−0.003	0.309	0.082	0.558	0.413	0.223
M4	0.082	0.087	0.167	1.206	−0.164	−0.030
F1	−0.286	−0.229	−0.004	0.504	0.007	−0.151
F2	0.078	−0.017	0.001	0.902	−0.248	−0.192
F3	0.269	−0.036	0.207	0.859	0.036	−0.108
F4	0.727	−0.153	−0.053	0.613	0.009	0.087

## Supplementary Materials

The following are available online at https://www.mdpi.com/1660-4601/18/5/2586/s1, Table S1: Descriptive statistics of the results of the survey, Table S2: Descriptive statistics of the results by countries, Figure S1: COMULATIVE DEATHS CASES DURING THE SURVEY (15–28 April 2020)
