# Supplementary material for: A Cross-Country Examination on the Fear of COVID-19 and the Sense of Loneliness during the First Wave of COVID-19 Outbreak
Source: Int J Environ Res Public Health. 2021 Mar 5;18(5):2586. doi: 10.3390/ijerph18052586 (PMC7967533; doi:10.3390/ijerph18052586)

Supplement table 1 Descriptive statistics of the results of the survey

	N	Fear of COVID-19 Mean, SD	Lack of companionship Mean, SD	Feeling left out Mean, SD	Feeling isolated Mean, SD
**Total Sample**	3876	13.62 (5.14)	2.31 (0.70)	1.56 (0.70)	1.88 (0.77)
Males	1422	12.57 (4.80)	2.25 (0.69)	1.45 (0.64)	1.75 (0.73)
Females	2442	14.23 (5.24)	2.34 (0.70)	1.62 (0.72)	1.95 (0.78)
Other	12	12.83 (4.47)	1.92 (0.79)	1.75 (0.97)	1.75 (0.87)
**Age**					
18-25 years (age1)	1885	12.81 (4.90)	2.40 (0.69)	1.57 (0.71)	1.94 (0.79)
26-39 years (age2)	954	14.00 (5.28)	2.26 (0.70)	1.50 (0.68)	1.83 (0.75)
40-59 years (age3)	881	14.50 (5.15)	2.18 (0.70)	1.58 (0.69)	1.79 (0.74)
> 59 years (age4)	156	16.08 (5.22)	2.22 (0.69)	1.58 (0.68)	1.85 (0.74)
**Country**					
Slovenia, Croatia, Serbia, Slovakia and Bosnia and Herzegovina **(C1)**	3066	13.06 (4.86)	2.34 (0.69)	1.59 (0.70)	1.91 (0.77)
Italy and Spain **(C2)**	810	15.72 (5.61)	2.17 (0.73)	1.45 (0.65)	1.75 (0.76)
**Gender & Age**					
males, age 1 (M1)	672	11.90 (4.57)	2.32 (0.68)	1.46 (0.64)	1.79 (0.75)
males, age 2 (M2)	378	12.67 (4.95)	2.22 (0.71)	1.42 (0.60)	1.75 (0.71)
males, age 3 (M3)	306	13.40 (4.82)	2.12 (0.69)	1.46 (0.64)	1.64 (0.70)
males, age4 (M4)	66	14.94 (4.77)	2.26 (0.69)	1.61 (0.72)	1.79 (0.73)
females, age 1 (F1)	1208	13.31 (5.01)	2.45 (0.68)	1.64 (0.73)	2.02 (0.79)
females, age 2 (F2)	570	14.87 (5.34)	2.29 (0.68)	1.56 (0.71)	1.89 (0.77)
females, age 3 (F3)	574	15.10 (5.22)	2.21 (0.70)	1.64 (0.73)	1.87 (0.75)
females, age 4 (F4)	90	16.91 (5.40)	2.20 (0.69)	1.57 (0.65)	1.89 (0.74)
**Gender & Country**					
males, countries 1 (MC1)	1014	11.74 (4.33)	2.30 (0.68)	1.47 (0.65)	1.77 (0.73)
males, countries 2 (MC2)	408	14.64 (5.23)	2.12 (0.72)	1.41 (0.61)	1.69 (0.73)
females, countries 1 (FC1)	2042	13.72 (4.98)	2.37 (0.69)	1.64 (0.72)	1.98 (0.78)
females, countries 2 (MC2)	400	16.84 (5.76)	2.23 (0.72)	1.49 (0.68)	1.82 (0.78)
**Age & Country**					
age 1, countries 1 (1C1)	1494	12.18 (4.58)	2.45 (0.67)	1.61 (0.72)	2.00 (0.79)
age 1, countries 2 (1C2)	391	15.19 (5.35)	2.23 (0.71)	1.42(0.62)	1.71 (0.73)
age 2, countries 1 (2C1)	693	13.20 (4.79)	2.28 (0.69)	1.51 (0.68)	1.84 (0.74)
age 2, countries 2 (2C2)	261	16.11 (5.90)	2.21 (0.70)	1.48 (0.67)	1.83 (0.77)
age 3, countries 1 (3C1)	751	14.22 (5.02)	2.22 (0.68)	1.60 (0.69)	1.80 (0.73)
age 3, countries 2 (3C2)	130	16.13 (5.59)	1.94 (0.75)	1.43 (0.65)	1.72 (0.79)
age 4, countries 1 (4C1)	128	15.74 (5.02)	2.24 (0.67)	1.59 (0.66)	1.85 (0.71)
age 4, countries 2 (4C2)	28	17.61 (5.90)	2.14 (0.76)	1.57 (0.79)	1.82 (0.86)
**Gender, Age & Country**					
males, age 1, countries 1 (M1C1)	471	10.81 (3.87)	2.38 (0.67)	1.49 (0.66)	1.84 (0.77)

males, age 1, countries 2 (M1C2)	201	14.46 (5.05)	2.18 (0.70)	1.38 (0.61)	1.67 (0.71)
males, age 2, countries 1 (M2C1)	242	11.76 (4.49)	2.26 (0.70)	1.42 (0.62)	1.75 (0.70)
males, age 2, countries 2 (M2C2)	136	14.31 (5.30)	2.14 (0.72)	1.42 (0.58)	1.76 (0.74)
males, age 3, countries 1 (M3C1)	248	13.02 (4.63)	2.17 (0.66)	1.46 (0.64)	1.65 (0.68)
males, age 3, countries 2 (M3C2)	58	15.05 (5.31)	1.90 (0.77)	1.45 (0.65)	1.57 (0.77)
males, age 4, countries 1 (M4C1)	53	13.96 (3.81)	2.32 (0.64)	1.62 (0.71)	1.75 (0.70)
males, age 4, countries 2 (M4C2)	13	18.92 (6.26)	2.00 (0.82)	1.54 (0.78)	1.92 (0.86)
females, age 1, countries 1 (F1C1)	1018	12.82 (4.74)	2.48 (0.67)	1.67 (0.74)	2.07 (0.79)
females, age 1, countries 2 (F1C2)	190	15.96 (5.55)	2.28 (0.73)	1.47 (0.64)	1.76 (0.76)
females, age 2, countries 1 (F2C1)	447	13.97 (4.79)	2.29 (0.69)	1.56 (0.70)	1.88 (0.76)
females, age 2, countries 2 (F2C2)	123	18.14 (5.94)	2.29 (0.67)	1.54 (0.74)	1.91 (0.80)
females, age 3, countries 1 (F3C1)	502	14.82 (5.10)	2.24 (0.69)	1.67 (0.71)	1.87 (0.74)
females, age 3, countries2 (F3C2)	72	17.00 (5.70)	1.97 (0.75)	1.42 (0.64)	1.83 (0.79)
females, age 4, countries 1 (F4C1)	75	17.00 (5.40)	2.19 (0.69)	1.56 (0.62)	1.92 (0.71)
females, age 4, countries 2 (F4C2)	15	16.47 (5.53)	2.27 (0.70)	1.60 (0.83)	1.73 (0.88)

Supplement table 2 Descriptive statistics of the results by countries

	N	Fear of COVID-19 Mean, SD	Lack of companionship Mean, SD	Feeling left out Mean, SD	Feeling isolated Mean, SD
Slovenia	445	14.57 (5.16)	2.27 (0.69)	1.63 (0.74)	1.93 (0.77)
Croatia	1646	12.28 (4.60)	2.39 (0.65)	1.59 (0.69)	1.88 (0.75)
Serbia	364	12.24 (4.62)	2.53 (0.63)	1.58 (0.69)	1.89 (0.78)
Slovakia	530	14.76 (4.76)	2.14 (0.78)	1.58 (0.73)	2.03 (0.81)
Bosnia and Herzegovina	92	12.82 (5.12)	2.37 (0.61)	1.38 (0.59)	1.71 (0.70)
Italy	512	16.27 (5.53)	1.79 (0.73)	1.28 (0.53)	1.62 (0.72)
Spain	313	14.87 (5.64)	1.79 (0.73)	1.28 (0.53)	1.62 (0.72)

Supplemental Figure

1. **Deaths:** How many deaths from Coronavirus have been reported in the period when survey was going on (from 15th to 28 of April 2020)?
2. **Cases:** How many cases were confirmed at that days, each day and cumulative?

	cumulative deaths				daily confirmed cases			comulative confirmed COVID-19 case		
	on 15 April 20	on 28 April 20	DELTA/during the survey	index	on 15 April 20	on 28 April 20	index	on 15 April 20	on 28 April 20	index
ITALY	21.069	26.977	5.908	28	2972	1739	-41,5	162488	199414	22,7
SPAIN	18.276	23.822	5.546	30	4424	1525	-65,5	182491	213095	16,8
SLOVENIA	56	83	27	48	8	0	-100,0	1220	1407	15,3
CROATIA	31	59	28	90	54	9	-83,3	1704	2039	19,7
SERBIA	94	162	68	72	411	233	-43,3	4465	8275	85,3
BOSNIA & HERCEGOVINA	39	60	21	54	45	48	6,7	1073	1564	45,8
SLOVAKIA	2	18	16	800	66	2	-97,0	835	1381	65,4
total	39.567	51.181	11.614	29	7980	3556	-55,4	354276	427175	20,6

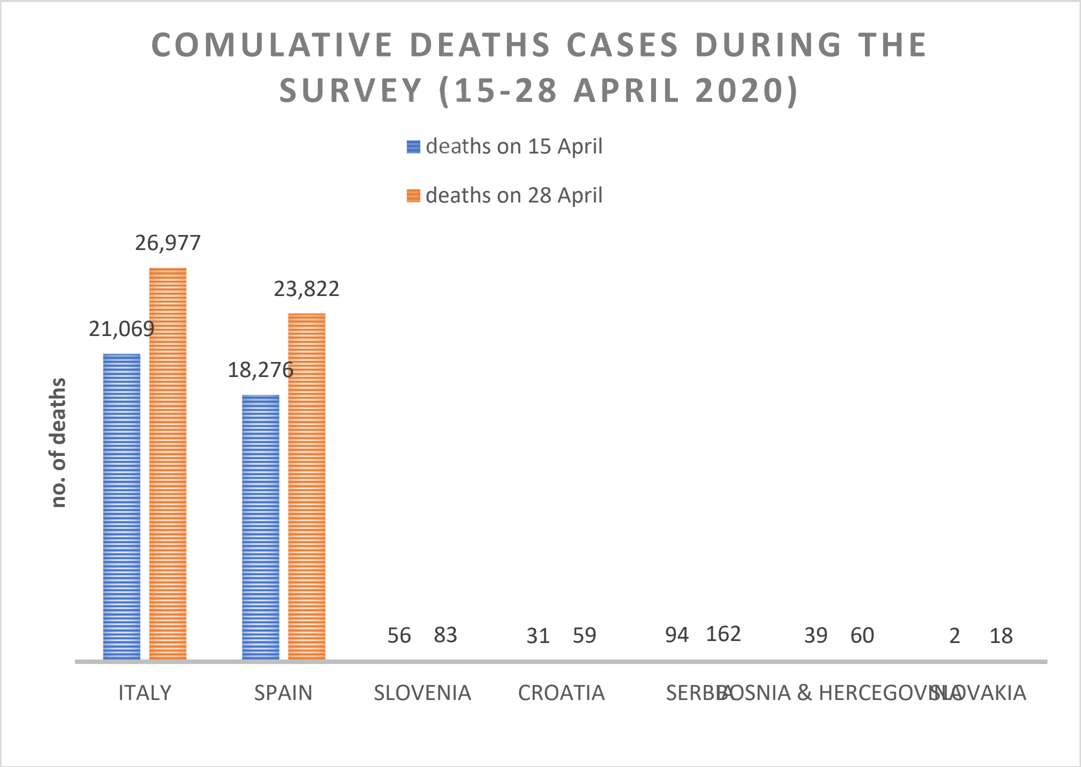

Supplement: Supplementary file 1 [file ijerph-18-02586-s001.pdf]
